# Chinese English as a Foreign Language Teachers’ Immunity and Mindfulness as Predictors of Their Work Engagement

**DOI:** 10.3389/fpsyg.2022.874356

**Published:** 2022-04-21

**Authors:** Shengji Li

**Affiliations:** Ural Institute, North China University of Water Resources and Electric Power, Zhengzhou, China

**Keywords:** Chinese EFL teachers, teachers’ success, immunity, mindfulness, work engagement

## Abstract

Considering the significant contribution of teachers’ professional triumph in the prosperity of students, the current study aims to investigate the existence of any relationship among three factors influencing teachers’ success: immunity, mindfulness, and engagement. Furthermore, we attempt to investigate whether English as a foreign language (EFL) teachers’ immunity and mindfulness can predict their work engagement. To this end, a Likert-scale questionnaire including items on teacher immunity, mindfulness, and work engagement was distributed to 582 EFL teachers in China through the WeChat application by employing a convenient sampling. To analyze the collected data, the Spearman Rho correlation index and linear multiple regression analysis are employed. The findings are that there does exist an indication of a direct relationship among EFL teachers’ immunity, mindfulness, and work engagement. Also, it is found that immunity and mindfulness can predict EFL teachers’ work engagement. The current study’s findings support the necessity of training language teachers to cope with the EFL context adversities.

## Introduction

As one of the concrete pillars of education (Pishghadam et al., 2021), teachers’ professional engagement and success is a determinant of the students’ academic achievement and the total effectiveness of the educational system (Mercer and Dörnyei, 2020). Typically teaching is considered as a challenging vocation and attention-intensive with uncertain nature (Crain et al., 2017), because it requires working with different learners and making hundreds of creative and flexible decisions on the run (Roeser et al., 2012). Recognized as a demanding and stressful occupation, teaching is usually accompanied by high levels of attrition, tension, and other inherently social-emotional demands but with low professional wellbeing (Benevene et al., 2020). However, most of the teachers are incredibly passionate and devoted to their profession. Schaufeli et al. (2002), considering this sense of commitment and zeal as work engagement, point out that enthusiastic teachers act energetically and are highly interested in what they do. In this respect, Cardwell (2011) argues that teachers’ high level of professional engagement favorably influences teaching quality.

Debate on providing access to highly engaged teachers is of critical importance in the recent educational context and research (Staiger and Rockoff, 2010; Pianta et al., 2012). A body of research has tried to study the effectiveness of various factors on teachers’ work engagement including content knowledge, technological pedagogical content knowledge (Han et al., 2020; Özgür, 2020; Bao et al., 2021; Beyranvand and Mohamadi Zenouzagh, 2021), teacher-efficacy and teacher autonomy (Skaalvik and Skaalvik, 2014), emotion regulation and resilience (Xie, 2021), and teacher intrinsic motivation (Bal and Bakker, 2010). To pursue this line of inquiry, the present study aims to examine the role of teacher immunity and mindfulness in predicting English as a foreign language (EFL) teachers’ work engagement in the Chinese context.

Language teacher immunity, as a potential predictor of teacher work engagement, introduced by Hiver and Dörnyei (2017), is a key constituent in the formation of language teachers’ behavior, perspective toward their profession, and professional identity (Hiver, 2015, 2016). Teacher immunity in general, and language teachers in particular, shed light upon the way teachers’ defense mechanism responds to and lessen the unfavorable impacts of commotion on EFL instructors’ occupational identity (Hiver and Dörnyei, 2017). Teachers’ experience of unrest like other psychological problems caused by challenges and problematic situations is one of the affective factors influencing their teaching. Such pressures mostly caused by L2 stakeholders can decline teachers’ immunity. Several studies have been done to explore the effect of different psychological factors on teacher immunity. For example, research on the effect of gender, age, and teaching experience (Ahmadi et al., 2020), job security, personality traits, (Rahimpour et al., 2020), reflective teaching (Rahmati et al., 2019). Since nobody is innately immune, teachers also are not inherently protected against unexpected and disturbing factors. Therefore, considering the importance of their profession, their immunity should be enhanced (Beyranvand and Mohamadi Zenouzagh, 2021). Training and equipping teachers with a crucial understanding of different emotional factors of the education context and emotion-regulation strategies can help them to succeed in their profession and consequently support and promote learners’ success (Bielak and Mystkowsks-Wiertelak, 2020; Derakhshan et al., 2021a).

Along with teacher immunity, mindfulness is another factor leading teachers to be engaged in their work. Mindfulness was defined as the ability to concentrate non-judgementally on purpose at the present time (Kabat-Zinn, 1994). In its operationalized definition, mindfulness refers to the self-regulation of attention on present events and the ability to be open and non-reactive. Therefore, mindfulness and awareness of the moment-to-moment experiences happening in the workplace can act as a promising shelter against stressors. Recent studies on mindfulness have shown that teachers’ familiarity with this concept can alter their understanding of attention-regulation and mindfulness, and consequently reduce job stress and distance from the profession (Jennings and Greenberg, 2009; Crain et al., 2017).

Considering the research on teacher immunity, mindfulness, and work engagement are in their infancy, the relationship among them has not received enough attention. Bakker et al. (2008) consider feeling energetic and absorbed by the work as the effective constituents of work engagement being relevant to teacher immunity. While several studies have been conducted on different factors influencing teacher engagement, particularly in the Chinese context (Hiver, 2017; Zeng et al., 2019; Han et al., 2020; Xie, 2021), the important variables of teacher immunity and mindfulness have received less attention. To our knowledge, few studies have been done on the relationship among immunity, mindfulness, and teachers’ absorbedness, i.e., work engagement, particularly in China. Therefore, this study aims to explore the relationship among these three factors and the prediction power of teacher immunity and mindfulness regarding Chinese EFL teachers’ work engagement in the Chinese EFL context by answering these research questions:

1. Is there any relationship among Chinese EFL teachers’ immunity, mindfulness, and work engagement?
2. Can the Chinese EFL teachers’ immunity and mindfulness predict their work engagement?

## Review of Literature

### Immunity

The concept of teacher immunity is a metaphor taken from its biological counterpart and refers to a type of defense or protective system used by individuals to handle the challenges and turbulences in their profession or life (Hiver, 2015). Similar to the body’s immune system’s function against external germs, the teacher immunity system operates as a ward to cope with stressors and setbacks in the language teaching context (Hiver, 2015).

As a new notion in teacher psychology, “Language teacher immunity” has been brought into the scene by Hiver (2015) during a study on defining the gaps between teachers’ professional identity and their motivation. With its two manifestations, productive and maladaptive, the teacher immunity system “bridges individual concerns with wider contextual considerations” (Hiver and Dörnyei, 2017, p. 407). According to Hiver and Dörnyei (2017), immunity is a double-edged sword that includes both sides of productive immunity and maladaptive immunity. These two manifestations center on how teachers succeed or fail in coping with hard knocks in their classroom and school settings. The productive form supplies language teachers with motivation, resilience, and enthusiasm to protect them in nerve-racking situations; while maladaptive representation makes teachers liable to pessimism, emotional exhaustion (Hiver and Dörnyei, 2017).

The teacher immunity construct is grounded on the self-regulation theories and processes, one of its core tenets, and the Complexity/Dynamic System Theory (CDST). Morrison (2002 cited in Li, 2021, p. 5) considers CDST as a “theory of change, evolution, and adaptation, which examines survival by a blend of cooperation and competition.” Hiver (2018) believes that teacher immunity is a type of self-regulation process: the gut “pattern-formation” and “change” in CDST. Particularly, as an adaptive process, the self-organization process involves changes in the function and internal structure of a system in response to disturbing factors to continue. This process entails following developmental phases: *triggering*, loss of balance in the system due to occurred adversities and becoming susceptible; *coupling*, the emergence of loops of positive feedback and interaction with other systems to strive against obstacles (Rahmati et al., 2019); *realignment*, restructuring itself and trying to attain stability and take back its productivity which brings about new patterns in the system; and finally, *stabilization*, the newly formed patterns in the behavior become a part of the system and stabilize (Hiver, 2016; Rahmati et al., 2019).

The type of taken immunity by the teachers, productive or maladaptive, is defined in the stabilization stage. Two recent studies investigated the process of developing immunity by Iranian EFL teachers showed that low motivation on the part of students and their parents’ high expectations, reflective teaching, low self-confidence, low income, and job security were the major disturbances triggering the teacher immunity (Haseli Songhori et al., 2018; Rahmati et al., 2019). According to their reports, among Iranian EFL teachers, maladaptive immunity was the outstanding type. Given that teacher immunity influences almost every dimension of teachers’ performance, it is recommended that investigating language teacher immunity and its predicting factors can provide a helpful and positive perception of teachers’ cognition, expertise, and identity (Hiver, 2015; Hiver and Dörnyei, 2017).

Reviewing the literature shows that the psychological concept of mindfulness has currently been the focus of the majority of research to be studied and evaluated for its efficacy. In this regard, different definitions and descriptions have been provided. As believed by Brown and Ryan (2003), mindfulness is an “inherently a state of consciousness” involving awareness of one’s real-time experience. In other words, it is an internal dynamic process enabling a human being to be aware of events and experiences around them. It armors the brain to contemplate and reflect on moment-by-moment experiences and thoughts (Hölzel et al., 2011). Roeser (2016) points out that mindfulness is a dynamic and changeable trait that can be mediated and trained. It is a non-judgmental awareness concerning the events in the present moment and interconnected with an individual’s peacefulness. In the educational setting, the term mindfulness alludes to the process of regulating emotions and training attention to assist teachers or students to focus only on the present time repentance and concern about the future and past (Ramasubramanian, 2017).

As a particularly demanding profession, teaching entails multifaceted functions of cognitive, emotional, and social competence (Jennings and Greenberg, 2009). Discussions regarding the required dispositions and qualities of a perfect teacher have led to an interest in studying and employing mindfulness-based approaches to education and teacher training contexts. The literature has shown that the best teachers have exceptional ability to track the activities in the classroom and simultaneously pay close attention to the whole class or one child. Being mindful of what is going on in the whole class and, at the same moment, being responsive to individual students’ needs require an uncanny ability to shift attention regularly between the whole class to individuals (Frank et al., 2016).

Along with this inclusion of *Observe* and *aware* dimensions, teachers need to focus attention on the studied subject matter ignoring their own or their students’ emotions. Working in such an emotionally challenging atmosphere, teachers need to make time to attentively scrutinize their emotional experiences to understand the origin of their emotional vulnerability and manage them (Jennings et al., 2011). A mindful teacher should be emotionally regulated, calm, clear, and stable in the face of challenges (Kabat-Zinn, 1994). Current studies signify the positive role of training mindfulness skills in improving teachers’ attention regulation and their perceptions of their mindfulness.

Furthermore, it can reduce job stress which has destructive consequences on the educational system as a whole and the EFL teaching context in particular (Fathi et al., 2021). Crain et al. (2017) believe that workplace mindfulness training helps to develop and exploit stress management skills such as awareness, focused attention, and a non-reactive attribute toward unpredicted experiences, whereby teachers can lessen their involuntary stress reactivity and reach a greater level of mental clarity and emotion calm.

### Work Engagement

As a motivational concept, work engagement with its integral conceptual dimensions, i.e., energy and involvement refers to the intentional commitment in the work (Christian et al., 2011; Han and Wang, 2021). Saks (2011) considers three cognitive, emotional, and physical domains for engagement. Klassen et al. (2013) hypothesized four dimensions for teacher engagement in their Engaged Teacher Scale (ETS): Physical, the devotion of a lot of energy; cognitive, absorbedness in the work while teaching; Emotional, putting heart into teaching; and finally Social including two aspects of social: students, well-connection with students; and Social: colleagues, being accessible to colleagues. According to Macey and Schneider (2015), work engagement indicates the inclination (sense of energy) leading to engaged deeds (acting energetically). Although reflecting motivational forces, engagement is conceptually different from them, job involvement and satisfaction (Rich et al., 2010), and work commitment which is a standpoint to work (Saks, 2006). Work engagement points to the amount of absorption and attention in the required tasks (Shuck et al., 2013). Operationalizing work engagement, Schaufeli et al. (2006) consider work engagement as a cognitive-affective state but ignore the social dimension.

Social engagement is one of the paramount dimensions of works engagement that characterize the teaching activity (Jennings and Greenberg, 2009). In the educational context, a level of energy is dedicated to establishing relationships, that is, demand for social engagement (Roorda et al., 2011). Unlike other working settings, social relationships constitute the heart of teaching and require spending energy to establish meaningful and long-term connections with students. The opportunity to establish relationships with students and work with them closely provides an intense motive for many teachers (Watt and Richardson, 2007). Furthermore, researchers believe that teacher-student connections may work as fostering source for student engagement and consequently positive outcomes (Wang, 2009). For instance, L2 students’ engagement in learning is positively correlated with teachers’ willingness to communicate (Mystkowska-Wiertelak, 2021), girt and emotions (Khajavy, 2021), and credibility behaviors (Derakhshan, 2021). On the other hand, the emergence of warm and supporting relationships between teacher and students lead teacher to experience a well amount of well-being as well as less burnout and stress (Jennings and Greenberg, 2009). The relationship between engagement and burnout is under debate. Regarding engagement as opposite to burnout, they are considered as the extremes of a continuum: low burnout (totally engaged) and high burnout (not engaged). Considering the scanty of research on factors affecting teacher engagement, more work should be done to conceive the fostering sources of teacher engagement in overall and EFL teachers in particular.

## Methodology

Based on Spearman Rho correlation index and linear multiple regression analysis, the present study employs a correlational (associational) design of research, since it can be used to test a relationship among variables as well as make predictions. To conduct the present work, Likert-scale questionnaires were used. As Mackey and Gass (2005) point out, questionnaires are “one of the most common methods of collecting data on attitudes and opinions from a large group of participants” p. 92).

### Participants

In this study, 582 Chinese EFL teachers participated in the study, whose ages ranged from 20 to 71. To generalize research results, both genders (male = 86, female = 496) were surveyed, and they mainly came from Henan Province (520/89%) and other provinces (Heilongjiang, Shanghai, Guangxi, Guangdong, Beijing, Hubei, Jiangsu, Sichuan, Shaanxi, Tianjin, and Zhejiang; 62/11%). Through convenience sampling, they participated in this research based on their willingness via WeChat using Wenjuanxing. Further demographic information is demonstrated in Table 1.

**TABLE 1:** Participants’ demographic information.

Background information	Number
**Gender**	
Male	86
Female	496
**Major**	
Applied linguistics	101
Linguistics	75
English language literature	143
English language translation	89
Other	174
**Last academic degree obtained**	
High school	1
Bachelor of arts	189
Master of arts	288
Ph.D.	32
Other	72
**Where are you currently teaching?**	
Public school	190
Private school	149
Public school and private school	10
Language institute	1
University	69
College	163
**Level of education you are currently teaching**	
Elementary school	100
Secondary school	38
High school	36
Higher education	404
Other	4
**Teaching experience**	
1–5	179
6–10	97
11–15	107
16–20	73
21–25	55
26–30	35
More	36

### Instruments

A Likert-scale questionnaire was employed to collect the data of the study. The validity of the questionnaires was evaluated by three experts and their reliability was estimated by Cronbach’s alpha. It is composed of three parts intending to investigate the participants’ perceptions of teacher immunity, mindfulness, and work engagement. The first part is a Consent form to ensure the participants of the confidentiality of obtained information and its academic use besides having their consent on voluntary participation. The second one asks about the demographic information including gender, age, nationality, major, last academic degree obtained, the country where the participants are currently teaching, their current place of teaching, level of education the participant’ are currently teaching, and teaching experience. The third part of the questionnaire comprises three sections of questions on teacher immunity, mindfulness, and engagement.

#### Teacher Immunity Scale

The teacher immunity scale includes thirty-nine Likert-scale items of the teacher immunity. They are scored on a 6-point frequency rating scale ranging from (strongly disagree) to (strongly agree). These items are *Resilience, Teaching Self-efficacy, Burnout, Attitudes Toward Teaching, Classroom Affectivity, Openness to Change, and Coping.*

#### Teacher Mindfulness Scale

The mindfulness questionnaire presents 14 statements describing how participants have felt at their work over the past 4 weeks indicating to what extent the statements are true, (Never true = 1,

Slightly true = 2, Moderately true = 3, almost true = 4, always true = 5), for them.

#### Teachers’ Work Engagement Scale

Finally, the Likert-scale items of work engagement measure the extent to which the participants are engaged in their job scored on a 7-point frequency rating scale.

### Data Collection Procedure

For data collection, questionnaires were distributed online through WeChat via Wenjuanxing, an online platform serving for collecting data. They spent 555 s finishing the questionnaires on average. A convenience sampling method was employed to obtain data from diverse parts of China and different Language teaching centers. The participants were all fully aware of their rights to withdraw from the study for any reason. Before beginning, they were informed of how to make validated answers and assured that their personal information was only used to meet the aims of this research. After checking the gathered responses for possible outliers, statistical analysis based on Chinese EFL Teachers’ Immunity, Mindfulness, and Work Engagement was conducted.

## Results

### Primary Data Analysis

In the initiation of the study, to make sure of the reliability of the instruments used for data collection, the researchers run a Cronbach’s alpha test for each questionnaire. Table 2 shows that all three questionnaires including teachers’ immunity, mindfulness, and work engagement have satisfactory Cronbach’s alpha indices (0.75, 0.77, and 0.96, respectively).

**TABLE 2:** Reliability of the questionnaires.

Questionnaires	Cronbach’s alpha	Number of items
Teacher immunity	0.749	39
Mindfulness	0.774	14
Work engagement	0.957	16

After making sure of the reliability of the questionnaires, a normality test is run to decide whether the data should be analyzed parametrically or not. Table 3 depicts that the collected data is not normal for all of the variables, since *P* values for them (teachers’ immunity, mindfulness, and work engagement) are 0.000. Thus, it violates the assumption of normality and the data has to be analyzed non-parametrically using a Spearman Rho correlation index.

**TABLE 3:** Test of normality.

	Kolmogorov–Smirnov^a^			Shapiro–Wilk		
	Statistic	df	Sig.	Statistic	df	Sig.
Immunity	0.246	582	0.000	0.819	582	0.000
Mindfulness	0.190	582	0.000	0.914	582	0.000
Engagement	0.241	582	0.000	0.893	582	0.000

*^a^Lilliefors significance correction.*

### The First Research Question

The first research question concerns the existence of any relationship among EFL teachers’ immunity, mindfulness, and work engagement in China. To this end, after making sure that the data is not normal, a Spearman Rho test is run.

Spearman Rho index shows the direction and the value of the relationship among the variables. Table 4 demonstrates that the relationship between teacher engagement and the other variables is direct, which means that the higher index of teacher engagement, the higher indices of the other variables. Furthermore, the significance level for all of these relationships is 0.000, that is, there is a direct and significant relationship among the variables of the study.

**TABLE 4:** Correlations among teachers’ immunity, mindfulness, and engagement.

			Immunity	Mindfulness	Engagement
Spearman’s rho	Immunity	Correlation coefficient	1.000	0.672**	0.597**
		Sig. (2-tailed)	.	0.000	0.663
		*N*	582	582	582
	Mindfulness	Correlation coefficient	0.672**	1.000	0.431**
		Sig. (2-tailed)	0.000	.	0.000
		*N*	582	582	582
	Engagement	Correlation coefficient	0.597	0.431**	1.000
		Sig. (2-tailed)	0.000	0.000	.
		*N*	582	582	582

**shows the significance of the value.

### The Second Research Question

The second research question investigates the extent to which Chinese EFL teachers’ immunity and mindfulness can predict their work engagement. To measure this prediction, a linear multiple regression analysis is performed.

The model summary Table 5 shows the amount of the variance in the dependent variable (scores obtained from work engagement) which can be clarified by the model (including the variables of teachers’ immunity and mindfulness). The obtained value is 0.44 (*R*^2^ = 0.44). Expressing in percentage, it suggests that the model (including scores on teachers’ immunity and mindfulness) explains 44% of the variance in scores from teachers’ work engagement.

**TABLE 5:** Model summary for teachers’ immunity, mindfulness, and engagement.

Model	*R*	*R* ^2^	Adjusted *R*^2^	Std. error of the estimate
1	0.666^a^	0.444	0.442	8.03

*^a^Predictors: (constant), mindfulness, immunity.*

Assessing the statistical significance of the results makes going over Table 6 indispensable. It examines the hypothesis that multiple *R* in the population equals zero (0). The model indicates statistical significance [*F* = (2, 579) = 231.35, Sig = 0.000, this really means *p* < 0.05].

**TABLE 6:** ANOVA for teachers’ immunity, mindfulness, and engagement.

Model		Sum of Squares	df	Mean Square	*F*	Sig.
1	Regression	29,853.60	2	14,926.80	231.35	0.000
	Residual	37,356.38	579	64.51		
	Total	67,209.99	581			

*^a^Dependent variable: engagement.*

*^b^Predictors: (constant), mindfulness, immunity.*

In order to understand the highly contributed variable in the prediction of work engagement, i.e., dependent variable, the researchers check the column labeled “Beta” in Table 7. It is felt necessary to consider the *standardized* coefficients, not the *unstandardized* ones when we aim to compare the different variables. “Standardized” refers to the converting of values related to different variables to the same scale with the aim of making them comparable.

**TABLE 7:** Coefficients for teachers’ immunity, mindfulness, and engagement.

		Unstandardized coefficients		Standardized coefficients	*t*	Sig.
Model		*B*	Std. error	Beta		
1	(Constant)	11.237	2.72		4.12	0.000
	Immunity	0.390	0.02	0.62	16.31	0.000
	Mindfulness	0.069	0.04	0.06	1.59	0.112

*^a^Dependent variable: engagement.*

In the current study, being interested to compare the role of each independent variable, we use the beta values. Considering the obtained Betas, we find that the highest beta coefficient was 0.62, which is for Teachers’ immunity. That is, teacher immunity makes the most significant contribution in relation to the existence of the dependent variable when the variance explained by all other variables in the model is controlled. The Beta value for the other variable (i.e., teaches’ mindfulness) is not significant since the Sig value is 0.112, which is more than 0.05.

## Discussion and Conclusion

The aim of the present study is to investigate the contribution of Chinese EFL teachers’ immunity and mindfulness in their engagement in the workplace. Therefore, this study has been done with two objectives: first, to examine the existence of any interrelationship among three psychological variables named teacher immunity, teacher mindfulness, and teacher work engagement; second, to examine the prediction power of teachers’ immunity and mindfulness regarding Chinese EFL teachers’ work engagement. Our findings are that there does exist an indication of a positive relationship among variables and the predictive power of teacher immunity and teacher mindfulness. The interrelatedness of these three factors has not been previously investigated in the EFL context of China. Considering the novelty of the teacher immunity, mindfulness, engagement, and lack of vast research coverage, the researchers try to get wise to new facts rather than supporting the previous studies.

Regarding the first research question, the results indicate that there is a direct positive correlation between teacher immunity, mindfulness, and work engagement. That is, EFL teachers who have a more powerful mindfulness system have a higher level of immunity and engagement and vice versa. This finding may unfold some factors affecting language teacher immunity and broaden our knowledge of the constructs that are inseparably linked with it. This part of the findings is consistent with Hiver’s statement of “teacher immunity affects almost everything that teachers do in their careers” (Hiver, 2015, p. 226).

Concerning the relationship between Chinese teacher immunity and their work engagement, the results reveal that there is a significant correlation. That is, teachers’ resistance to workplace challenges and staying in an energetic manner in their profession are related to commitment and engagement. This finding confirms Hiver (2017) who believes that there is a reciprocal relationship between engagement and immunity and explains it in relation to resilience and self-efficacy as the integral constituents of teacher immunity. Hiver (2017) argues that one of the contributing factors to language teacher immunity is resilience helping them to remain engaged and to cope with the challenges. Considering language teaching as fundamentally relationship-based, Hiver (2018) and Pishghadam et al. (2021) believe that teacher resilience can be cultivated in the teacher-student interactions bringing about finding purpose on the part of the teacher and consequently engaging in meaningful teaching. According to the study by Han et al. (2020) in the Chinese context, teacher efficacy has a positive correlation with teacher engagement. In this respect, our findings support Noughabi et al. (2020) who pointed out that “various aspects of teacher engagement (i.e., cognitive engagement, emotional engagement, social engagement with learners, and social engagement with colleagues) could affect experienced EFL teachers’ immunity” (p. 7). It can be claimed that engagement can help teachers to pave the way to reach productive immunity. Therefore, supporting the earlier reporting of a positive association between teacher engagement and efficacy and resilience in China (Han et al., 2020; Bao et al., 2021), the reports from the data analysis of the present study indicate that teacher engagement and teacher immunity are tied together. However, the relationship of engagement and other constituents of teacher immunity such as coping, classroom affectivity, openness to change, attitudes toward teaching, and Burnout needs to be studied.

As concerning the relationship between teacher mindfulness and immunity, and engagement, the current study showed a positive direct relationship. Although there is no research on the relationship of these three variables, each as a whole construct, there are some correlational studies on their constituent. This finding supports Chiesa et al. (2011) who argue that mindfulness practices can promote teachers’ regulation strategies and psychological potentials, whereby teachers’ substantiality increases. According to previous studies, the mindfulness of teachers broadens their social relationships, which we can consider as social engagement, self-care, reflectivity (Li, 2021). Furthermore, the correlational studies pinpoint a close tie between mindfulness and task engagement (Kee and Liu, 2011), teacher wellbeing (Jennings, 2015), emotional regulation (Bielak and Mystkowsks-Wiertelak, 2020), and teachers’ and students’ academic performance (Li, 2021). A study by Waldman and Carmel (2019) on the impact of mindfulness on L2 teachers’ self-efficacy shows that there is a positive correlation between mindfulness practice and self-efficacy. Since the relationship between immunity and mindfulness is direct and positive, it supports the idea that mindfulness can minimize automatic stress reactivity and increase mental clarity and emotional calm as well as the ability to cope and openness to change (Crain et al., 2017).

As to the second research question and the prediction power of the teacher immunity and mindfulness concerning Chines EFL teachers’ work engagement, the finding shows that immunity and mindfulness can predict the existence of engagement. Considering the direct and positive correlation among these three variables, finding this predictive value seems reasonable. Since the previous studies (Hiver, 2017; Noughabi et al., 2020) and also the findings of research question one show a positive and reciprocal relationship among teacher immunity, mindfulness, and work engagement, the prediction of work engagement by the level of immunity and mindfulness is deductible. In addition to obtaining evidence on prediction power, we are interested to know which variable can be a more powerful predictor. The analysis of the data shows that EFL teachers’ immunity contributes highly to Chinese EFL teachers’ engagement in the workplace. To sum up, the review of the literature suggests that there are scant studies on them and more complementing research is demanded in different contexts.

In light of working on the present manuscript, these types of research have valuable implications for stakeholders in education and particularly EFL contexts. It is clear that teachers’ affect, emotions, and psychology play a critical role in education whose performance is influenced by many external and internal factors. Hence, students’ motivation, learning, and achievements all depend on the teacher’s motivation (Derakhshan et al., 2021b), and other psychological states. Given that teaching is a demanding and stressful occupation placing tensions and pressures on teachers, they must be trained and psychologically prepared to cope with such adversities. This line of research can be beneficial for teachers in that they get aware of the language teaching demands and the fact that this profession is famous for its emotional strains, adversities, and stress (Derakhshan, 2021). Similarly, by learning how to deal with these adversities through developing required strategies teachers can create an affluent atmosphere for students. On the other hand, the familiarity of students with the language teaching and learning nature, and their responsibility in creating a rich instructional setting, they make an effort to minimize classroom tensions to help their teacher feel relaxed and focus on the teaching subject.

General to the research world, our study also suffers from some flows which can be eliminated in future studies. It is mentionable that the primary limitation of our work was that our data were collected under the shadow of the COVID-19 pandemic. Although the participants were asked to consider “normal times” in answering the questionnaire, not during the pandemic, it is supposed that far-working has its own tensions and adversities which can influence EFL teachers’ psychological perceptions and contaminate the data. The second limitation is related to the online data collection and dispersing of the questionnaires. Due to the pandemic constraints on traveling, dispersing questionnaires in-person to EFL teachers from different parts of China was impossible and most of our participants came from one province which influence the generalizability of the findings. Furthermore, collecting data from EFL teachers with different demographic characteristics, especially their teaching experience and the level they teach couldn’t consider in detail. Finally, employing a mere questionnaire may shed light on some aspects and others remain in shadow. Therefore, it is suggested to include an interview and even retrospective methods after some workdays in future studies to have a clear understanding of the emerged relationships among immunity, mindfulness, and work engagement. Besides, considering the existence and also the director of the relationship among the studied variables is suggested in EFL online classes and in real normal time, respectively. Last but not least, we suggest the replication of the present study in different teaching levels, i.e., in schools, language institutes, and higher education, and on teachers with different years of experiences separately to have the effect of experiences and teaching levels on the relationship of EFL teachers’ immunity, mindfulness, and engagement in their work.

## Data Availability Statement

The raw data supporting the conclusions of this article will be made available by the author, without undue reservation.

## Ethics Statement

The studies involving human participants were reviewed and approved by NCWU Academic and Ethics Committee. The patients/participants provided their written informed consent to participate in this study.

## Author Contributions

The author confirms being the sole contributor of this work and has approved it for publication.

## Conflict of Interest

The author declares that the research was conducted in the absence of any commercial or financial relationships that could be construed as a potential conflict of interest.

## Publisher’s Note

All claims expressed in this article are solely those of the authors and do not necessarily represent those of their affiliated organizations, or those of the publisher, the editors and the reviewers. Any product that may be evaluated in this article, or claim that may be made by its manufacturer, is not guaranteed or endorsed by the publisher.
